# Computational Biology: A New Frontier in Applied Biology

**DOI:** 10.3390/biology10050374

**Published:** 2021-04-27

**Authors:** Milan Toma, Riccardo Concu

**Affiliations:** 1 Serota Academic Center (Room 138), New York Institute of Technology, Department of Osteopathic Manipulative Medicine, College of Osteopathic Medicine, Northern Boulevard, P.O. Box 8000, Old Westbury, NY 11568, USA; 2 Faculty of Science, University of Porto, Rua do Campo Alegre, s/n, 4169-007 Porto, Portugal

All living things are related to one another. Hence, the science of biology is comprehensive in scope. *Biology*, i.e., studying the structure, function, growth, origin, evolution, and distribution of living organisms is a vast and eclectic field. The science of biology is divided into different branches. Some divisions define up to 25 different branches, e.g., cell biology, genetics, immunology, molecular biology, biotechnology, biophysics, biomathematics, anatomy, etc. Needless to say, computational biology is not one of the branches. While computational biology largely relies on biomathematics, mathematics alone can be divided into pure versus applied. Hence, we refuse to link computational biology with the biomathematics branch of biology directly. Any of the 25 different branches can be studied both experimentally and computationally. Computational algorithms and models serve to extend our understanding of how organisms work from subcellular to whole organism level (Figure 1).

From a single organelle to the entire biosphere, living organisms are parts of a highly structured hierarchy. Some cells contain aggregates of macromolecules surrounded by membranes, called organelles (articles [1,2,3,4] in this special issue fall under this category). All living things are made of cells [5,6,7,8]. In larger organisms, cells combine to make tissues [9]. Organs are collections of tissues grouped together, performing a standard function [10,11,12,13]. Organisms are individual living entities. All the individuals of a species living within a specific area are collectively called a population [14]. An ecosystem consists of all the living things together with the non-living parts of that environment. The biosphere is the collection of all ecosystems. Furthermore, again, every entity in this hierarchy can be studied using computational algorithms and models. This special issue contains articles contributing to almost every entity (see individual references in Figure 1). The 14 articles, totaling 314 pages, in this issue, have been co-authored by 74 researchers from 12 countries.

Not long ago, biologists did not have access to vast amounts of data. Recent advancements in technology are enabling us to generate and store an incredible amount of data. However, our ability to decipher data is slower than our ability to generate them. Thus, an increasing amount of unknown data is stacking up in databases such as the protein data bank. Due to this, by framing biomedical problems as computational problems, using tools adapted from computer science, mathematics, statistics, physics, chemistry, and other quantitative disciplines, scientists use those data to develop analytical methods, algorithms, and models for interpreting biological information. However, achieving comprehensive predictive models of biological systems requires these models to be understandable and reproducible. Unfortunately, a few existing models are reproducible, as we lack the data sources utilized and assumptions/equations used to build the models. To address these issues, the Center for Reproducible Biomedical Modeling aims to “accelerate the development of comprehensive predictive models by enhancing the understandability, reusability, and reproducibility of biomedical modeling”.

To standardize computational techniques used to assess the safety of medical devices, a worldwide benchmark study, namely the FDA’s “Critical Path” project, has been organized. The benchmark flow model used for that study consisted of a nozzle with a concentrator and sudden expansion, see the schematic in Figure 2. Over 40 groups (self-ascribed as beginner, intermediate, or expert) delivered their results. The graph in Figure 2 shows the best and worst fitting results from each group compared to experimental results from three labs. The results of the FDA study show that the computational results need to be validated even when produced by experts. It can be seen, in Figure 2, that even the worst ‘beginner’ is better than the worst ‘expert’. A rigorous approach to the development of complex modeling software packages is necessary. The modeling and analysis software packages must be reliable.

A review of computational biology has to include the pioneer of the field, Alan Turing (23 June 1912–7 June 1954). He is celebrated as the godfather of modern computing. However, what remains a little known fact about his work is his contribution to the field of biology. Turing studied morphogenesis—the biological process by which organisms take their shape. He developed one of the first mathematical models of how biological shapes emerge. Turing published only one paper on morphogenesis 2 years before his death, and more of his work on the subject was published posthumously in the third volume of his collected works. Turing defined some of the mathematical rules that govern biology, and with that, he assumed that there were similarities between how machines and biological organisms compute. There is much more work to be done in exploring such questions.

## Figures and Tables

**Figure 1:**
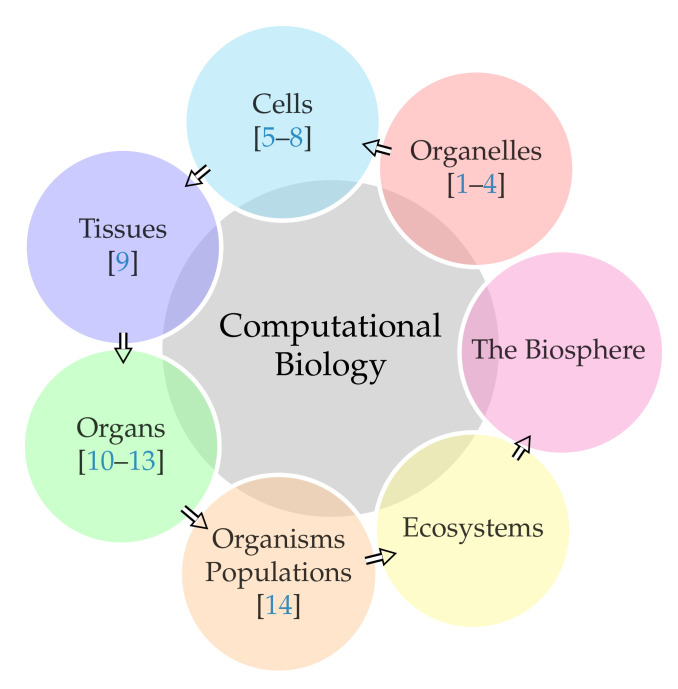
From a single organelle to the entire biosphere, living organisms are parts of a highly structured hierarchy.

**Figure 2:**
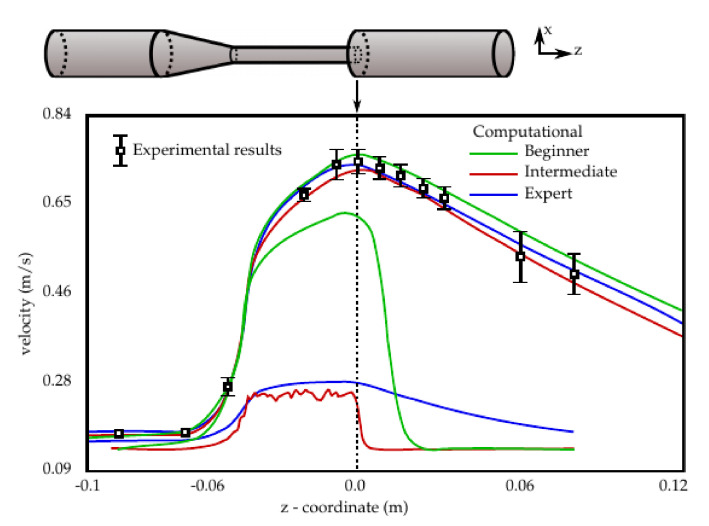
Results from the FDA’s “Critical Path” project to validate computational methods. The best and worst fitting results are shown from each (self-ascribed) category.
